# Cardiac Autonomic Neuropathy as a Result of Mild Hypercaloric Challenge in Absence of Signs of Diabetes: Modulation by Antidiabetic Drugs

**DOI:** 10.1155/2018/9389784

**Published:** 2018-01-31

**Authors:** Ola Al-Assi, Rana Ghali, Ali Mroueh, Abdullah Kaplan, Nahed Mougharbil, Ali H. Eid, Fouad A. Zouein, Ahmed F. El-Yazbi

**Affiliations:** ^1^Department of Pharmacology and Toxicology, Faculty of Medicine, The American University of Beirut, Beirut, Lebanon; ^2^Department of Pharmacology and Toxicology, Faculty of Pharmacy, Alexandria University, Alexandria, Egypt

## Abstract

Cardiac autonomic neuropathy (CAN) is an early cardiovascular complication of diabetes occurring before metabolic derangement is evident. The cause of CAN remains elusive and cannot be directly linked to hyperglycemia. Recent clinical data report cardioprotective effects of some antidiabetic drugs independent of their hypoglycemic action. Here, we used a rat model receiving limited daily increase in calories from fat (HC diet) to assess whether mild metabolic challenge led to CAN in absence of interfering effects of hyperglycemia, glucose intolerance, or obesity. Rats receiving HC diet for 12 weeks showed reduction in baroreceptor sensitivity and heart rate variability despite lack of change in baseline hemodynamic and cardiovascular structural parameters. Impairment of cardiac autonomic control was accompanied with perivascular adipose inflammation observed as an increased inflammatory cytokine expression, together with increased cardiac oxidative stress, and signaling derangement characteristic of diabetic cardiomyopathy. Two-week treatment with metformin or pioglitazone rectified the autonomic derangement and corrected the molecular changes. Switching rats to normal chow but not to isocaloric amounts of HC for two weeks reversed CAN. As such, we conclude that adipose inflammation due to increased fat intake might underlie development of CAN and, hence, the beneficial effects of metformin and pioglitazone.

## 1. Introduction

Diabetes mellitus is a metabolic disorder characterized by hyperglycemia caused by reduced secretion or action of insulin or both [1]. Two main forms of diabetes exist, type 1 and type 2. Type 1 diabetes is an autoimmune disease characterized by the destruction of pancreatic *β*-cells with concomitant insulin deficiency and resistance. Type 2 diabetes encompasses insulin resistance and deficiency and accounts for 90–95% of diagnosed patients worldwide [1]. According to the 2014 World Health Organization (WHO) Global Report on Diabetes, the prevalence of diabetes is on the rise. About 422 million people have diabetes worldwide, and it is estimated that approximately 1.5 million died of a cause related to diabetes in 2012 [2]. The increased prevalence is thought to be triggered by changes in human behavior, lifestyle, and environment [3]. Rapid dietary changes towards high saturated fat, high sugar, refined, and low in fiber foods contributed to the increase in diabetes and cardiovascular diseases [3].

Cardiovascular complications are considered the major and primary cause of morbidity and mortality in diabetes, where eight out of ten diabetic patients die because of cardiovascular diseases [4]. While prediabetic patients are considered to be in an intermediate metabolic state that does not fulfill the diagnostic criteria of diabetes, studies found that they remain at an increased risk of cardiovascular disease [5, 6]. Indeed, a significant proportion of newly diagnosed diabetic patients had established microvascular complications at initial presentation [7] indicating that pathological stages early in diabetes development constitute an important window for potential interventions to alleviate the detrimental sequelae of diabetes.

Recent studies detected early changes in cardiovascular autonomic control in diabetic patients before other signs of peripheral and autonomic neuropathies manifest [8, 9]. Cardiac autonomic neuropathy (CAN) represents a main cause of mortality in diabetic patients, where it is associated with a high risk of sudden death and cardiac arrhythmia [10]. The prevalence of diabetic CAN could reach up to 34% of type 2 diabetic patients [11]. Despite its documented negative impact on cardiovascular outcomes in type 2 diabetes patients [12], it remains as one of the least understood, recognized, and studied complications of diabetes [10]. While gross neuronal damage due to metabolic derangement could account for CAN in diabetic patients [10], the same cannot be considered for prediabetic patients [13]. Metabolic syndrome hallmarks, other than hyperglycemia, were implicated in the development of CAN in this patient cohort including obesity, triglyceride derangement, and insulin resistance [14].

Similar to other cardiovascular complications of diabetes, strong clinical data suggest that tight glycemic control can slow the progression and the development of autonomic dysfunction [15]. For the longest time, no data were available to describe a preferential positive outcome of using a particular therapeutic approach for glycemic control. Yet, recent clinical evidence from cardiovascular outcome trials [16, 17] suggests that treatment with a given molecule might provide protection against cardiovascular events in diabetic patients even when careful attempts are made to keep HbA1C% under close control. Of particular significance, favorable cardiovascular effects of antihyperglycemic drugs apart from their effect on glycemic control were observed in prediabetic patients treated by pioglitazone [18] and metformin [19] raising the question of whether these drugs could interfere with the early detrimental cardiovascular pathology prior to development of diabetes.

In this context, previous studies from our laboratory [20] showed that structural and functional vascular and hemodynamic alteration occurred prior to the development of hyperglycemia in a rat model of hypercaloric intake. The observed vascular dysfunction was reversed upon treatment with metformin or pioglitazone without changes in serum glucose level or advanced glycation end products. Significantly, multiple lines of evidence implicated increased caloric intake in the form of high-fat diets in increased central neuroinflammation prior to the development of signs of diabetes [21, 22]. Interestingly, not only are metformin and pioglitazone reported to have direct, glycemic-independent anti-inflammatory effects [23, 24], but also clinical evidence showed they possessed neuroprotective effects in patients with metabolic syndrome [25] and were effective in reducing CAN in diabetic patients [26]. As such, in the present study, we used a rat model of hypercaloric intake to examine whether CAN could develop as a consequence of mild metabolic challenge in the absence of hyperglycemia and peripheral markers of insulin resistance and cardiovascular involvement. We tested whether the determining factor was the overall caloric intake versus increased proportion of saturated fat in diet and if treatment with metformin and pioglitazone could potentially reverse the observed dysfunction.

## 2. Methods

### 2.1. Ethical Approval

All animal experiments were done according to an experimental protocol approved by the Institutional Animal Care and Use Committee in compliance with the Guide for Care and Use of Laboratory Animals of the Institute for Laboratory Animal Research of the National Academy of Sciences, USA.

### 2.2. Experimental Design

Male Sprague-Dawley rats (5-6 weeks of age; 150 g) were randomly divided into six groups (8 rats per group): (1) rats fed with normal chow diet (control, 3 Kcal/g), (2) rats fed with mild hypercaloric diet (HC, 4.035 Kcal/g) for 12 weeks, (3) rats fed with mild hypercaloric diet and treated with 100 mg/kg metformin at week 10 (Met), (4) rats fed with mild hypercaloric diet and treated with 2.5 mg/kg pioglitazone at week 10 (Pio), (5) rats fed with mild hypercaloric diet for 10 weeks, then switched to normal chow for the remaining 2 weeks (NC), (6) rats fed with mild hypercaloric diet ad libitum for 10 weeks, then switched to pair feeding with the same diet to match calorie intake of control rats on normal chow (HC-L). Normal chow diet (ENVIGO) was obtained from Teklad Rodent Diets (Madison, WI). This diet offers 3 Kcal/g distributed as follows: 32% from protein, 14% from fat (0.9% saturated fat by weight), and 54% from carbohydrates. The HC diet was prepared in house through the addition of food grade fructose (20% by weight, Santiveri foods, Spain) and hydrogenated vegetable oil (Mazola®, 15% by weight, BFSA). Major electrolytes and vitamins were supplemented to match the concentration in ENVIGO diet and as recommended by the American Institute of Nutrition [27]. The final composition of the HC diet by weight (calorie content) is 18.06% fat (38.68%, 5% saturated fat by weight), 15.8% protein (15.66%), and 46.13% carbohydrates (45.73%). Other than rats in group 6, all rats had free access to food and water for the full 12-week duration. Rats were kept in a temperature- and humidity-controlled room, in a 12-hour light/dark cycle. Body weight was measured weekly along with the anus-to-nose length (ANL), and calorie intake was calculated daily based on the amount of food consumed. At week 10, the treatment regimen was administered to different groups by daily oral gavage. The control groups received water gavage while drug treatment groups received freshly prepared aqueous suspensions of the antidiabetic agents.

### 2.3. Noninvasive Blood Pressure Measurement and Echocardiography

Rat blood pressure was measured noninvasively by tail cuff using CODA High Throughput Monitor (Kent Scientific, Torrington, CT) [28]. Measurement was performed at weeks 0, 4, 10, and 12. Any irregular or unacceptable recording noted as a false recording by the system was excluded. The parameters obtained are systolic (SBP), diastolic (DBP), mean arterial blood pressure (MAP), and heart rate (HR). Echocardiography was performed in the parasternal long axis M-mode and B-mode to monitor heart function and visualize heart morphology using SonixTouch Q+ ultrasound (BK ultrasound, Peabody, MA) [29]. Measurements were performed at weeks 0, 4, 10, and 12. The parameters calculated were left ventricular diastole diameter (LVDd), left ventricular posterior wall diameter (LVPWd), left ventricle mass (LV mass), end diastolic volume (EDV), and ejection fraction (EF).

### 2.4. Random Blood Glucose Measurement and Oral Glucose Tolerance (OGT) Testing

Random glucose testing was performed at weeks 0, 4, 8, and 12. The lateral tail vein was pricked, and an enough quantity of blood was obtained. The measurement was done using Accu-Chek Performa glucometer (Roche Diagnostics, Basel, Switzerland). At the end of week 12, rats were fasted overnight. Rats were challenged using a 2 g/kg, 20% glucose solution administered by oral gavage [30]. They were gently restrained, and blood glucose was measured at 0, 15, 30, 60, and 120 minutes after glucose load. Blood was collected from a tail vein prick, and glucose was measured using Accu-Chek Performa glucometer.

### 2.5. Invasive Hemodynamic Recording in Anesthetized Rats

At the end of the treatment period, rats were instrumented for arterial pressure and heart rate recording as described previously [31]. Briefly, following anesthesia using 100 mg/kg phenobarbital (AUB-MC pharmacy), tracheostomy was performed and right carotid artery was isolated, cannulated, and connected to a Millar transducer to measure mean arterial pressure (MAP) and heart rate (HR). Data acquisition was performed by LabChart Pro 8 (AD Instruments Ltd., Dunedin, New Zealand). Afterwards, the left jugular vein was isolated, cannulated, and connected to a shunt for IV drug administration. The recording was allowed to stabilize for 30 minutes, and baseline invasive SBP, DBP, MAP, and HR were recorded.

Baroreceptor sensitivity (BRS) was assessed using the vasoactive method [32]. Increasing doses of phenylephrine (PE, 0.25, 0.5, 0.75, 1, and 2 *μ*g) and sodium nitroprusside (SNP, 1, 2, 4, and 8 *μ*g) were administered, and the changes in MAP and HR were recorded. Slope of the linear regression fit between ΔHR and ΔMAP at different PE and SNP doses was used as a measure of BRS. Heart rate variability (HRV) analysis was performed using the available software module as described previously [33]. Cardiac autonomic activity was assessed using two time domain and two frequency domain measures. Variables were estimated as a five-minute average through an area of a stable recording pattern. The standard deviation of beat-to-beat intervals (SDNN) and the root mean square of successive beat-to-beat differences in R-R interval durations (rMSSD) were assessed as measures of the overall autonomic balance and parasympathetic input to the heart, respectively [34–36]. Frequency domain analysis was performed using Fast Fourier Transform algorithms of R-R data series. Spectra were integrated into 2 specific frequency bands, low frequency (LF, 0.25–0.75 Hz) and high frequency (HF, 0.75–3 Hz) bands, corresponding to sympathetic and vagal outflows, respectively [36], and power spectral density was determined for these two bands.

### 2.6. Histopathology and Immunohistochemistry

At the end of the invasive hemodynamic recording, rats were decapitated. Brain was dissected, placed on ice, and the brainstem was isolated. The thoracic cavity was exposed, pericardiac and aortic perivascular adipose tissues were dissected, and the heart was flushed and isolated. Adipose tissue was flash frozen in liquid nitrogen and stored at −80°C. Brainstems were cut in half; one side fixed in formaldehyde while the other was stored at −80°C. Ventricles were horizontally cut into 3 sections (apex, midsection, and base). The midsection was placed in formaldehyde for further histological analysis. Other parts were flash frozen in liquid nitrogen and stored at −80°C. For histopathology and immunohistochemistry, ventricular midsections and fixed brainstems were embedded in paraffin, sectioned transversely, and placed on clean slides. Staining was performed simultaneously for accurate comparison. For demonstration of nucleus and cytoplasmic inclusions, hematoxylin and eosin stains were used, for the detection of cardiac fibrosis, Masson trichrome staining was performed, while for estimation of reactive oxygen species activity, dihydroethidium staining was performed on cryosectioned heart midsection and brainstem as described previously [37]. For detection of transforming growth factor-*β* (TGF-*β*), sections were incubated with the primary antibody (rabbit anti-TGF*β*, 1 : 100, Abcam, Cambridge, UK) and detected using Novolink Polymer Detection Kit (Leica Biosystems, Buffalo Grove, IL) according to the manufacturer's protocol.

### 2.7. Western Blotting

Protein extraction and Western blotting were performed according to a method we previously developed and optimized [33, 38–40]. Briefly, the heart and brain tissue samples kept at −80°C were crushed under liquid nitrogen. 10 mg heart tissue and 40 mg brainstem tissue were transferred to 1 ml and 300 *μ*l, respectively, of a protein extraction buffer containing 1% sodium dodecylsulfate (SDS), 0.9% NaCl, 80 mM Tris hydrochloride (pH 6.8), and 100 mM dithiothreitol. Tissue amounts used for protein extraction were optimized according to final protein concentration in preliminary experiments. Samples were heated at 95°C for 10 min, allowed to cool down and transferred to a rocking shaker and left overnight for protein extraction at 4°C. Aliquots with equal protein content from the extracts were then used for SDS-PAGE and blotted as described previously [38]. After transfer and fixation, nitrocellulose membranes were blocked with 5% skim milk (Bio-Rad, Hercules, CA, USA) in Tris-buffered saline containing 0.1% Tween 20 for two hours at room temperature. At this stage, membranes were cut at the appropriate molecular weights to allow for probing of multiple proteins within the same run. Membranes were incubated in a dilution of primary antibodies in 1% skim milk in 0.1% TBST (1 : 1000 for rabbit polyclonal anti-AMPK*α*, rabbit polyclonal P-AMPK*α*, and P-Erk1/2, Cell Signaling Technologies, Danvers, MA, and rabbit polyclonal anti-Erk1/2, Thermoscientific, Waltham, MA, and 1 : 500 for rabbit polyclonal anti-interleukin (IL-1*β*), Abcam, Cambridge, UK) overnight at 4°C. After washing with 0.05% TBST (4 × 5 min), membranes were incubated in 1 : 10,000 biotin-conjugated goat anti-rabbit Ig for 1 h at room temperature followed by washing and incubation with 1 : 100,000 horse radish peroxidase-conjugated streptavidin for 30 minutes at room temperature. Blots of the brainstem samples were developed using the traditional two-step Western blotting using 1 : 5000 horse radish peroxidase-conjugated goat anti-rabbit secondary antibody in 0.1% TBST for one hour at room temperature. After washing 4 × 5 min with 0.05% TBST and 2 × 5 min with TBS, membranes were exposed to Clarity Western ECL substrate (BioRad, Hercules, CA) for 5 min following image detection using ChemiDoc imaging system (BioRad, Hercules, CA). Band optical density was measured using ImageJ software, and a ratio of arbitrary density units was obtained for the protein band of interest and the density of the band representing total protein for p-AMPK and p-Erk1/2 after stripping and reprobing, while IL-1*β* bands were normalized to actin as described previously [33, 38] to correct variabilities in loading and sample concentration.

### 2.8. Determination of IL-1*β* and Tumor Necrosis Factor (TNF-*α*) Expression in Perivascular Adipose Tissue by Quantitative Polymerase Chain Reaction (Q-PCR)

Q-PCR was carried out as previously described [41]. Briefly, total RNA was extracted from adipose tissue using an RNeasy Mini kit with DNase treatment (Qiagen, Hilden, Germany) and first strand cDNA synthesized using the Sensiscript RT kit (Qiagen, Hilden, Germany) with oligo d(T) primer. Primer pairs to detect rat IL-1*β* and *β*-actin were designed and purchased from Sigma (St. Louis, MO). The primer sequences (5′–3′) were the following: IL-1*β* forward GCCTCAAGGGGAAGAATCTATACC, reverse GGGAACTGTGCAGACTCAAACT; TNF-*α* forward ACCACGCTCTTCTGTCTACTG, reverse CTTGGTGGTTTGCTACGAC; *β*-actin forward GTCAGGTCATCACTATCGGCAAT and, reverse AGAGGTCTTTACGGATGTCAACGT. Primer sets used had an efficiency of >90% that did not differ by >5% at the annealing temperature and produced a single peak with no evidence of additional amplicons or primer dimer formation during melt curve analysis. Q-PCR was performed with SYBR Green and a reaction with a hot start at 95°C for 5 min, followed by 40 cycles of 95°C for 15 s, 60°C for 1 min, and 72°C for 1 min. Threshold cycle was determined using a Bio-Rad iCycler (Hercules, CA) and vendor-supplied software, and transcript abundance was calculated by the 2^−ΔΔCt^ method using *β*-actin as the reference for normalization.

### 2.9. Chemicals

All chemicals were obtained from Sigma (St. Louis, MO) unless otherwise indicated. Pharmaceutical grade metformin HCl, phenylephrine, and pioglitazone were obtained as kind gifts from regional pharmaceutical manufacturers (Merck, Pharo Pharma, and Hikma Pharmaceuticals).

### 2.10. Statistical Analysis

Data were expressed as mean ± SEM. Comparisons between groups were done using one-way ANOVA followed by Dunnett post hoc test, as well as two-way ANOVA followed by Sidak's multiple comparisons test in comparing different time points or doses among groups using Graphpad Prism software. *P* value < 0.05 was considered statistically significant.

## 3. Results

### 3.1. Calorie Intake, Weight Variation, and Blood Glucose Levels

**Figure 1:**
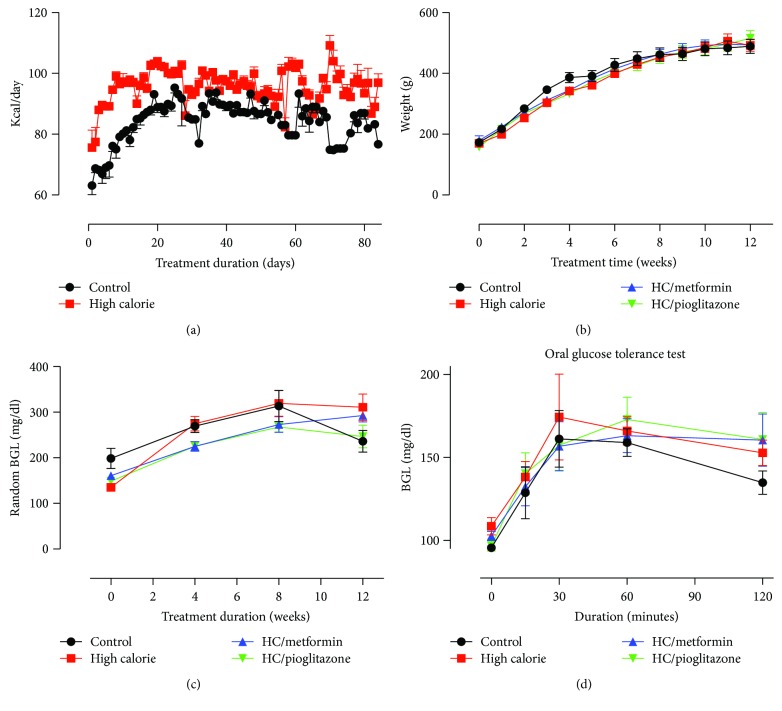
Daily calorie intake, body weight variation, and changes in blood glucose levels as a function of time in different treatment groups. (a) HC-fed rats (red) consumed higher calories on a daily basis compared to control rats on normal chow (black). Depicted data are mean ± SEM for eight control and 32 HC-fed rats. Daily calorie intake was significantly higher on most treatment days, *P* < 0.05 as estimated by two-way ANOVA. (b) No differences in weight gain were detected among the different treatment groups over twelve weeks of treatment. (c) No significant differences in random blood glucose levels were detected among the different treatment groups over twelve weeks of treatment. (d) At the end of the treatment period, different groups responded similarly to an oral glucose load. Data depicted in (b–d) represent mean ± SEM of values obtained from eight rats in each treatment groups.

On a daily basis, HC-fed groups showed an increased energy intake of ~14 Kcal compared to rats on normal chow (*P* < 0.05 on most days, Figure 1(a)). Energy intake gradually increased for both groups and then plateaued three weeks into treatment. For the remainder of experimental period, the HC group consumed ~100 Kcal/24 hours, while the NC group intake continued at ~86 Kcal/24 hours.

After initial randomization, rats from all groups had approximately the same weight (172.7 g ± 4.94). Rats from different groups continued to gain weight similarly throughout the experiment (Figure 1(b)). BMI was calculated using ANL and showed no significant difference between different groups (data not shown). Weight change within each group in the last 2 weeks during the treatment was determined. Only rats receiving pioglitazone treatment showed significant increase in body weight when compared to control and HC rats (data not shown).

As for blood glucose levels, HC feeding did not induce any changes in random blood glucose levels through the first 10 weeks of treatment (Figure 1(c)). Treatment with metformin or pioglitazone produced no significant change in random blood glucose in week 12. By the end of week 12, no significant differences in OGT were found amongst the four treatment groups (Figure 1(d)).

### 3.2. Noninvasive Blood Pressure Measurement

**Figure 2:**
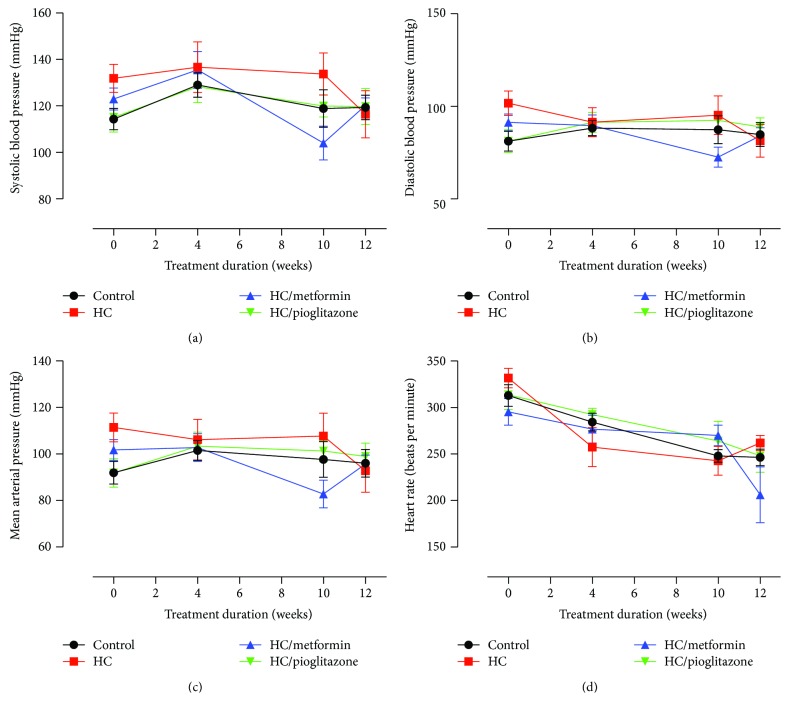
Noninvasive hemodynamic parameters of different treatment groups. No significant differences were detected among the different treatment groups in SBP (a), DBP (b), MAP (c), or HR (d) at baseline, 4, 10, and 12 weeks of treatment. Depicted data represent mean ± SEM of values obtained from eight rats in each treatment groups.

Chronic feeding with HC did not induce detectable changes in blood pressure throughout the duration of the experiment.  Figure 2 shows SBP (2a), DBP (2b), and MAP (2c) measured by tail cuff technique at different time points. Additionally, no significant differences were observed in HR recorded using the CODA monitor (2d). As well, rats receiving metformin or pioglitazone showed no significant difference in these parameters in the last two weeks.

### 3.3. Echocardiography

**Figure 3:**
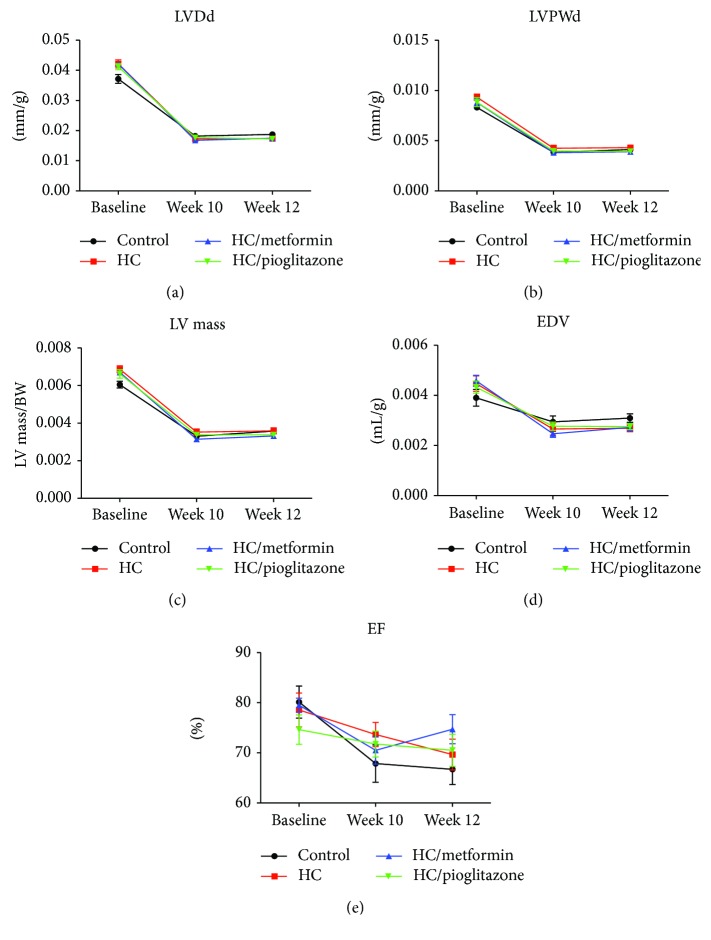
Echocardiographic parameters representing structural and functional aspects of the left ventricle. No significant differences were detected among the different treatment groups in LVDd (a), LVPWd (b), LV mass (c), EDV (d), or EF (e) at baseline, 10 and 12 weeks of treatment. Depicted data represent mean ± SEM of values obtained from eight rats in each treatment groups. All represented parameters are normalized to body weight (BW).

Echocardiography parameters calculated were left ventricular diastole diameter (LVDd), left ventricular posterior wall diameter (LVPWd), end diastolic volume (EDV), left ventricular mass (LV mass), and ejection fraction (EF). These parameters reflect the morphological (LVDd, LVPWd, and LV mass) and functional (EDV and EF) cardiac properties.  Figure 3 depicts changes in these parameters normalized to body weight. No significant differences in echocardiography parameters were noticed among different treatment groups. However, these parameters showed an apparent rapid decrease in all groups in the early treatment phase. This is caused by the normalization of the parameters against the rat body weight, where the rate of increase in the parameters measured was much less than the rate of increase in body weight.

### 3.4. Invasive Blood Pressure Measurement

At the conclusion of the 12-week treatment period, rats from different groups were instrumented for invasive hemodynamic recording. After 30 minutes of stabilization, a stable five-minute recording was used to calculate the invasive SBP, DBP, MAP, and HR. Similar to noninvasive measurements, no significant differences were detected in the baseline parameters of all treatment groups (data not shown).

### 3.5. Baroreceptor Sensitivity (BRS)

**Figure 4:**
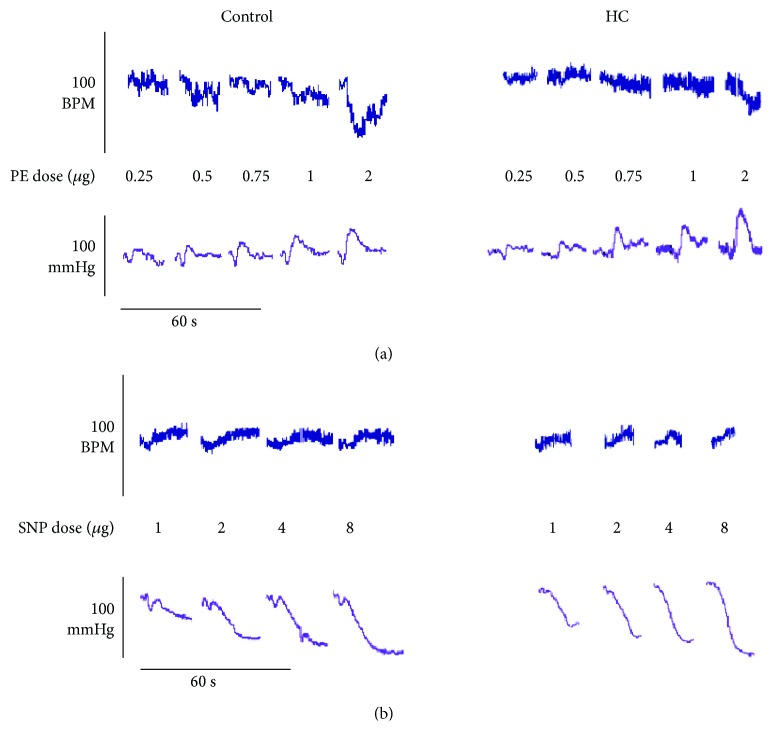
Representative tracings of the MAP and HR response of control (left) and HC-fed (right) rats to different doses of PE (a) and SNP (b). Vertical scale bars represent HR and MAP as indicated while horizontal scale bars represent time (60 s).

**Figure 5:**
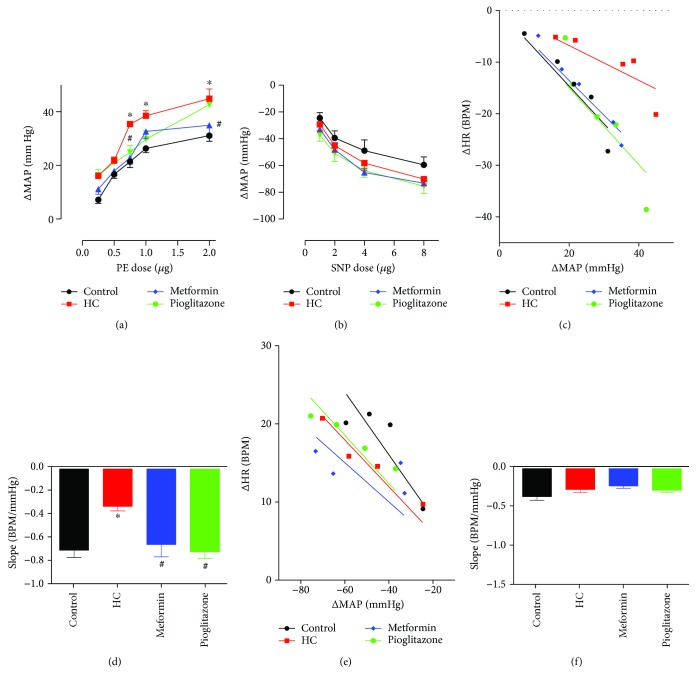
The effect of HC feeding and treatment with metformin or pioglitazone on BRS. (a) The pressor effect of different doses of PE in different treatment groups. ∗ and # denote *P* < 0.05 versus response at corresponding PE doses in control or HC-fed rats, respectively. Statistical significance was determined by two-way ANOVA followed by Sidak's post hoc test. (b) The reflex bradycardic response to different doses of PE in different treatment groups. (c) Best fit regression lines for the correlation between changes in MAP in response to increasing PE doses and reflex change in HR in different treatment groups. (d) Slope of the best fit regression line of the ΔMAP versus ΔHR relationship representing BRS in response to PE treatment. ∗ and # denote *P* < 0.05 versus slope in control or HC-fed rats, respectively. Statistical significance was determined by ANOVA followed by Dunnett post hoc test. (e) Best fit regression lines for the correlation between changes in MAP in response to increasing SNP doses and reflex change in HR in different treatment groups. (f) Slope of the best fit regression line of the ΔMAP versus ΔHR relationship representing BRS in response to SNP treatment. Depicted data represent mean ± SEM of values obtained from eight rats in each treatment groups.

After the equilibration period, a series of PE and SNP doses were administered and the resulting change in MAP (ΔMAP) and HR (ΔHR) was recorded.  Figure 4 depicts representative tracings of the changes in MAP and HR in response to PE (4A) and SNP (4B). An exaggerated vasopressor response to PE was noted in the HC group (Figure 5(a)). A trend towards a reduction of the increased vasopressor response was evident for rats treated with metformin or pioglitazone that was statistically significant at 0.75 *μ*g PE. No significant differences were detected in ΔHR in response to different PE doses among different treatment groups (Figure 5(b)). BRS was determined by the linear regression of ΔMAP versus ΔHR curves (Figure 5(c)). HC blunted BRS as seen in the reduced BRS line slope that was significantly lower than that of the control group. The recovery of BRS was significant in metformin- and pioglitazone-treated rats.  Figure 5(d) represents the statistical comparison of the regression line slopes. Neither ΔMAP nor ΔHR in response to SNP appeared to show significant differences among the different groups (data not shown). As well, there were no differences in BRS in response to SNP (Figure 5(e)) as can be inferred by the lack of statistical significance upon comparison of the slopes (Figure 5(f)).

### 3.6. Heart Rate Variability (HRV) Analysis

**Figure 6:**
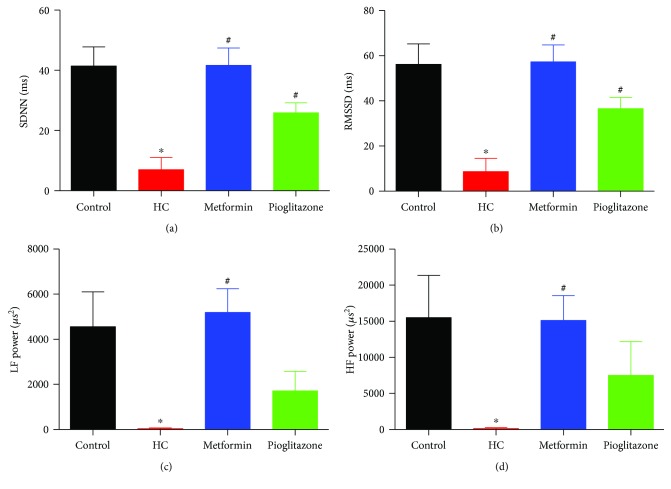
Changes in time domain and frequency domain HRV parameters in different treatment groups. (a), (b), (c), and (d) represent changes in SDNN, rMSSD, power spectral density of LF, and power spectral density of HF, respectively, among different treatment groups. ∗ and # denote *P* < 0.05 versus corresponding values in control or HC-fed rats, respectively. Statistical significance was determined by ANOVA followed by Dunnett post hoc test. Depicted data represent mean ± SEM of values obtained from eight rats in each treatment groups.

Following the equilibration period, a stable 15-minute recording of arterial pressure was analyzed for determination of time domain and frequency domain parameters.  Figure 6(a) represents SDNN while Figure 6(b) represents rMSSD. Both time domain parameters were significantly reduced in HC rats. Groups treated with metformin or pioglitazone showed significant recovery in both parameters. HC group had an abolished power spectral density in LF and HF compared to the control group (Figures 6(c) and 6(d)). Treatment with both metformin and pioglitazone restored both powers to a level not different from that of the control group.

### 3.7. Microscopic, Oxidative Stress, and Signaling Protein Expression/Phosphorylation Changes in Ventricular Tissue

**Figure 7:**
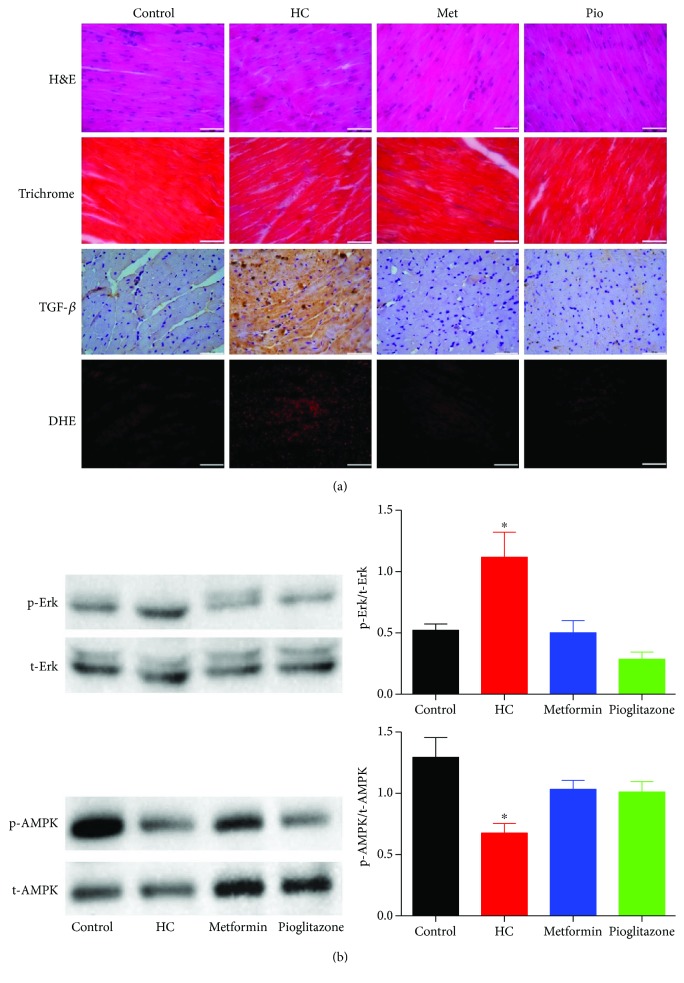
Cellular and molecular changes in ventricular tissue in response to HC feeding and the effect of treatment with metformin or pioglitazone. (a) Representative micrographs of histopathological staining, TGF-*β* immunostaining, and DHE staining of ventricular midsection. Data presented are serial sections obtained from the same tissue and are representative of tissues harvested from four rats in each group. Scale bars are 50 *μ*m. (b) Changes in phosphorylation of Erk1/2 (above) and AMPK (below) in rat ventricles in response to HC feeding and treatment with metformin or pioglitazone. The depicted blots are representative of experiments performed on protein extracts from tissues harvested from four rats in each group. The bar graphs represent comparison of the normalized intensity of the phosphorylated protein bands. ∗ denotes *P* < 0.05 versus corresponding values in control rats. Statistical significance was determined by ANOVA followed by Dunnett post hoc test.

Figure 7(a) shows representative micrographs of ventricular midsection stained with H&E and trichrome, immunostained for TGF-*β*, in addition to cryosections exposed to DHE staining. In line with the lack of change in echocardiography parameters representing cardiac structure/function, H&E and trichrome staining showed normal myocyte arrangement and distribution without a noticeable increase in collagen deposition in sections from HC-fed rats compared to control rats. However, in agreement with the previous data from our laboratory [20], TGF-*β* staining was increased in HC-fed rats and decreased with metformin or pioglitazone treatment. In addition, DHE staining showed an increased reactive oxygen species in tissue sections from HC-fed rats, which was reduced by treatment with metformin or pioglitazone.

On the other hand, multiple lines of evidence suggest that AMP-activated protein kinase (AMPK) phosphorylation is reduced in early stages of diabetic cardiomyopathy [42], whereas extracellular signal-regulated kinase (Erk1/2)/TGF-*β* signaling pathway is activated. AMPK and Erk1/2 phosphorylation levels were determined in ventricular tissue by Western blotting. Examination of ventricular protein extract showed reduced and increased phosphorylation of AMPK and Erk1/2, respectively, in tissues from HC rats (Figure 7(b)). These changes in protein phosphorylation were reversed following treatment with metformin or pioglitazone.

### 3.8. Changes in Oxidative Stress and Signaling Protein Expression/Phosphorylation in Brainstem

**Figure 8:**
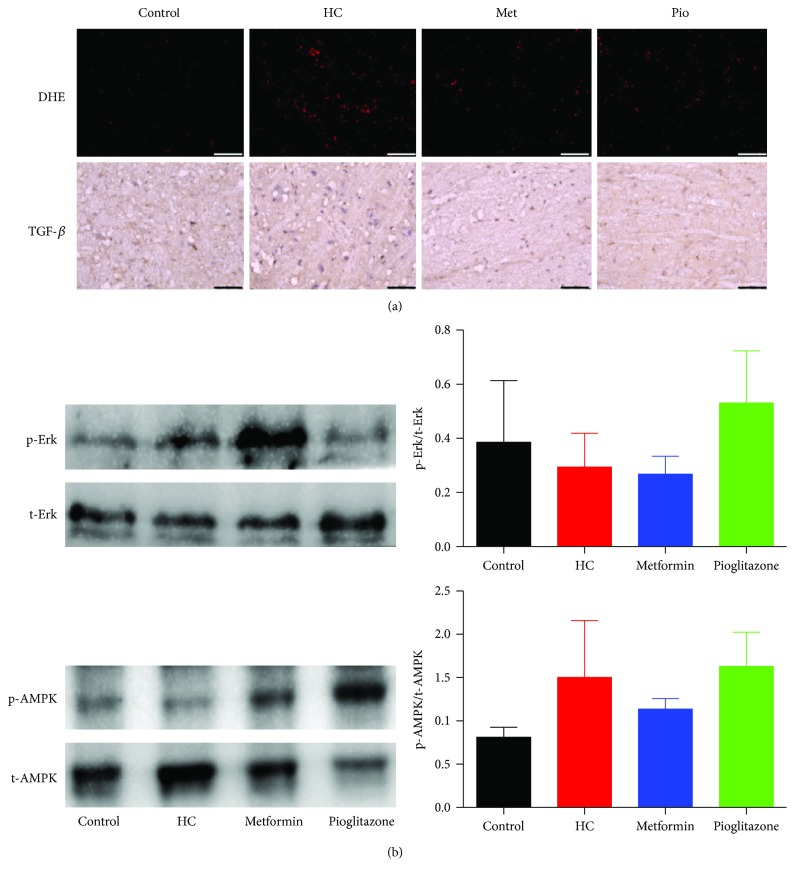
Cellular and molecular changes in brainstem in response to HC feeding and the effect of treatment with metformin or pioglitazone. (a) Representative micrographs of DHE and TGF-*β* immunostaining of brainstem sections. Data presented are serial sections obtained from the same tissue and are representative of tissues harvested from three rats in each group. Scale bars are 50 *μ*m. (b) Changes in phosphorylation of Erk1/2 (above) and AMPK (below) in brainstem in response to HC feeding and treatment with metformin or pioglitazone. The depicted blots are representative of experiments performed on protein extracts from tissues harvested from three rats in each group. The bar graphs represent comparison of the normalized intensity of the phosphorylated protein bands.

The effect of HC feeding and drug treatment on central neuronal sites in the brainstem controlling cardiac autonomic function was examined. The level of oxidative stress was assessed by DHE staining of cryosections from brainstem obtained from rats in different treatment groups. Compared to control rats, HC feeding increased reactive oxygen species in brainstem as can be seen in increased DHE staining intensity (Figure 8(a)). A trend towards a slight reduction in oxidative stress levels was observed in brainstem sections obtained from rats treated with metformin and pioglitazone; however, DHE staining remains noticeably higher than the control in these sections. Significantly, no changes were observed in TGF-*β* staining in brainstem sections from HC-fed rats compared to control (Figure 8(a)), neither were there changes in AMPK and Erk1/2 phosphorylation levels detected by Western blotting (Figure 8(b)).

### 3.9. The Effect of Drug Treatment on Adipose, Heart, and Brainstem Inflammatory Markers

**Figure 9:**
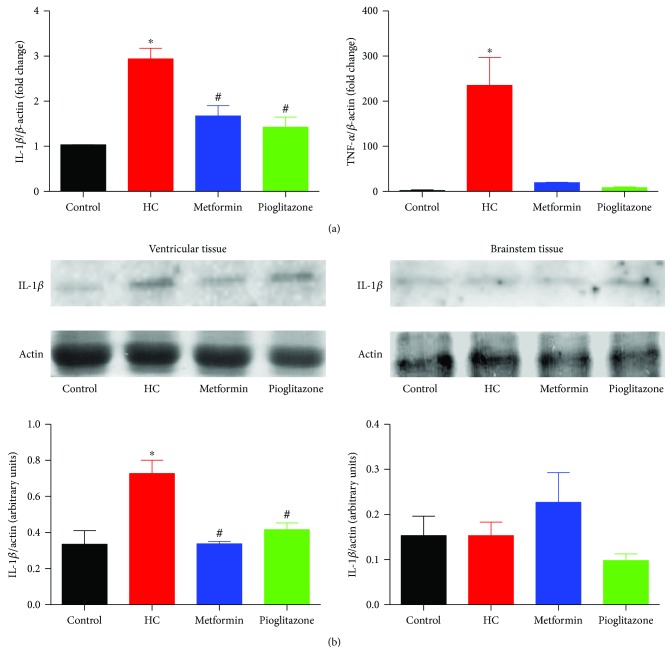
The effect of HC feeding and treatment with metformin or pioglitazone on inflammatory cytokine expression in perivascular adipose, ventricular, and brainstem tissues. (a) Changes in IL-1*β* (left) and TNF-*α* (right) mRNA levels in perivascular adipose tissue. Values were determined in triplicates of mRNA extracts from five different animals. (b) Representative Western blots showing changes in IL-1*β* expression levels in ventricular tissue (right) and brainstem tissue (left) in response to HC feeding and metformin or pioglitazone treatment. The depicted blots are representative of experiments performed on protein extracts from tissues harvested from three rats in each group. The bar graphs represent comparison of the normalized intensity of the protein bands. ∗ and # denote *P* < 0.05 versus corresponding values in control or HC-fed rats, respectively. Statistical significance was determined by ANOVA followed by Dunnett post hoc test. Depicted values represent mean ± SEM.

Several lines of evidence implicated IL-1*β* in the pathogenesis of high-fat diet-induced inflammation together with increased expression in the adipose tissue in human obesity and insulin resistance [43]. As well, increased TNF-*α* mRNA levels were observed in adipose tissue from humans with early metabolic dysfunction [44] and rats fed a fat-rich diet [45]. As such, we examined the effect of HC feeding on IL-1*β* and TNF-*α* mRNA levels in perivascular and pericardiac adipose, together with IL-1*β* expression in the heart and brainstem. As shown in Figure 9, in line with the previous findings, HC feeding increased IL-1*β* and TNF-*α* expression levels in adipose tissue and in ventricular extracts. Significantly, similar to TGF-*β* expression and AMPK and Erk1/2 phosphorylation, no change in IL-1*β* expression was observed in brainstem tissue. Subsequent treatment with metformin or pioglitazone was associated with a reduction in IL-1*β* transcript and protein levels in adipose tissue and ventricular extracts, respectively, supporting a possible reduction of adipose inflammation in these treatment groups.

### 3.10. The Effect of Dietary Switching on the Observed Changes in Cardiac Autonomic Control

**Figure 10:**
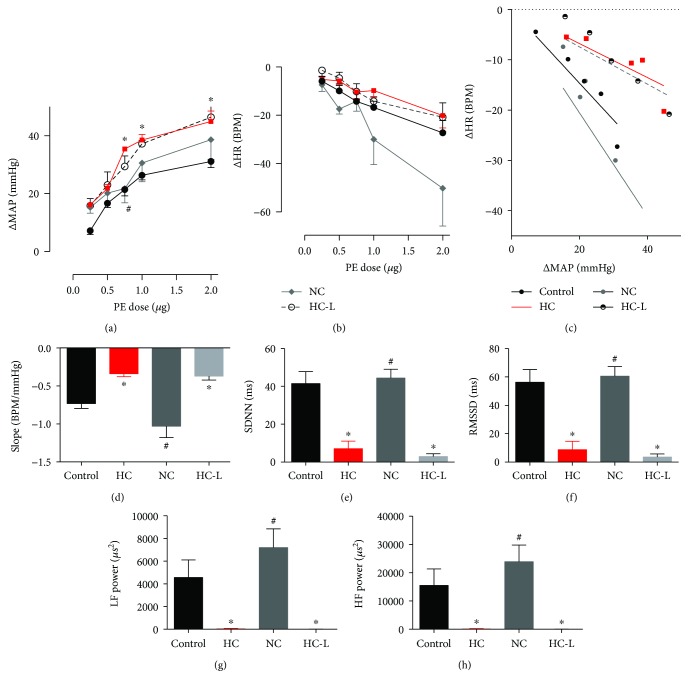
The effects of switching to normal chow (NC) versus isocaloric feeding with HC (HC-L) on BRS and HRV. (a) The pressor effect of different doses of PE. ∗ and # denote *P* < 0.05 versus response at corresponding PE doses in control or HC-fed rats, respectively. Statistical significance was determined by two-way ANOVA followed by Sidak's post hoc test. (b) The reflex bradycardic response to different doses of PE. (c) Best fit regression lines for the correlation between changes in MAP in response to increasing PE doses and reflex change in HR. (d) Slope of the best fit regression line of the ΔMAP versus ΔHR relationship representing BRS in response to PE treatment. (e) and (f) represent changes in time domain parameters of HRV, SDNN (e), and rMSSD (f). (g) and (h) represent changes in the power spectral density of LF (g) and HF (h). Data depicted represent mean ± SEM of values obtained from eight rats in control and HC groups and five rats in NC and HC-L groups. ∗ and # denote *P* < 0.05 versus slope in control or HC-fed rats, respectively. Statistical significance was determined by ANOVA followed by Dunnett post hoc test.

Previous literature implicated adipose inflammation and hypothalamic inflammatory signaling as consequences of high-fat feeding [46, 47], as opposed to increased calorie intake per se. In order to assess whether the observed deficit in cardiac autonomic control resulted from increased caloric intake versus an increased saturated fat proportion in diet, the effect of switching to normal chow (NC) or pair feeding with an isocaloric amount of our HC diet (HC-L) for the last two weeks on BRS and HRV was examined. Increased sensitivity to the vasopressor effect of PE persisted only in the HC-L group whereas a reduction similar to the effect obtained with metformin and pioglitazone treatments was seen in the NC group (Figure 10(a)). No significant changes were seen in HR (Figure 10(b)), and as such, BRS remained attenuated in HC-L but recovered in the NC group (Figures 10(c) and 10(d)). No differences were observed in the responses to SNP (data not shown). In parallel, deficits in time domain (Figures 10(e) and 10(f)) and frequency domain (Figures 10(g) and 10(h)) parameters of HRV persisted and recovered in the HC-L and NC groups, respectively.

## 4. Discussion

In the present study, we assessed whether cardiac structural and functional deterioration starts early in the course of metabolic alteration and examined the possibility of reversing these deleterious effects. To our knowledge, this is the first report of CAN occurring in the context of a metabolic challenge in the absence of detectable signs of obesity, hyperglycemia, or insulin resistance. As well, our present results highlight a potential involvement of adipose inflammation and a possible corrective role for metformin and pioglitazone that is not related to their blood glucose-lowering effect. In order to prove this association, hypercaloric intake in rats was used as a model of mild metabolic challenge. This model receives 38% of energy intake as fat, which is slightly higher than the American Diabetes Association daily total fat intake recommendations (20–35% energy as fat) [48] but within range of high-fat diet compositions (20–60% energy as fat) reported in the literature and used in validated rat models of obesity, insulin resistance, and diabetes [49]. While the increased dietary fat is the source of elevated calorie intake, a dietary composition containing fructose was chosen to simulate typical Western diets rich in refined sugars and saturated fat previously shown to be linked to cardiovascular pathologies in both rats and humans [50]. Fructose is particularly enriched in prepared foods, corn syrup additives, and carbonated beverages [51, 52]. Additionally, a recent comprehensive study [30] showed that, in contrast to high fat only, a combination of high-fat and high-fructose feeding on long term (8 months) led to the development of a more robust type 2 diabetes phenotype. Previous studies report that diets containing high fat alone and those with a combination of high fat/high fructose have similar detrimental effects on cardiovascular function in rats [30, 53–55]. In our previous studies [20], this diet was associated with the development of stable fasting hyperglycemia after 16 weeks of treatment and thus provided a wider window for examination of potential molecular, structural, and functional changes occurring early in the course of metabolic challenge without interference from hyperglycemia or obesity.

No significant differences were noted among the control and HC groups in baseline BP and HR, random blood glucose levels, and OGT. Up to twelve weeks of treatment and prior to invasive examination and sacrifice, echocardiography revealed neither structural nor functional cardiac changes. Upon assessing cardiac autonomic control, multiple functional differences became apparent indicating a detrimental effect of high-calorie intake and a potential corrective effect of metformin, pioglitazone, and dietary interventions involving reduction of saturated fat intake. This detrimental effect was further corroborated by the observations of adverse molecular changes at the level of the myocardium (increased oxidative stress and TGF-*β* expression, decreased AMPK phosphorylation, and increased Erk1/2 phosphorylation), adipose tissue (increased inflammatory cytokine expression), and brainstem (increased oxidative stress).

Significantly, when compared to the control group, the HC group had an exaggerated vasopressor response to PE and blunted BRS and HRV. The increased response to PE is in line with the previous findings from our laboratory and from other groups showing an increased sensitivity to contractile agonists observed in isolated vessel preparations from rats receiving high-fat diet [20, 56, 57]. BRS alteration only in response to PE-driven changes in MAP implied that HC-fed rats had a reduction in vagal control but maintained integrity of sympathetic regulation. Alongside, reduction in SDNN signified a reduction in overall cardiac autonomic control and this was confirmed by the concomitant reduction in HF power (parasympathetic) and LF power (sympathetic). These results are in agreement with the previous findings in diabetic, prediabetic, and metabolic syndrome patients showing disrupted HRV parameters [58–61]. Of note, despite the lack of apparent functional or microscopic myocardial deficit, changes seen in TGF-*β* and IL-1*β* expression, oxidative stress, and AMPK and Erk1/2 phosphorylation imply an early molecular insult that might potentially lead to cardiac structural involvement over time. Indeed, reduced AMPK phosphorylation was observed in type 2 diabetic rat hearts and thought to contribute to cardiac metabolic remodeling and left ventricular dysfunction [42]. Moreover, the activation of TGF-*β*/Erk1/2 pathway was reported to contribute to increased fibrosis observed in the context of diabetic cardiomyopathy [42, 62]. As such, our treatment model represents the early cardiac molecular deterioration in the context of metabolic challenge preceding the development of diabetes.

From a central autonomic control perspective, recent work demonstrated that high-fat feeding was associated with increased oxidative stress in brain tissue that was linked to poor cognitive function [63]. In our model, we examined changes in the brainstem as the site for autonomic cardiovascular and vasomotor centers. CAN induced by HC feeding was associated with an increased oxidative stress. Indeed, a significant body of evidence links increased ROS production in brainstem to the autonomic dysregulation seen in cardiovascular disorders such as neurogenic hypertension [64]. On the other hand, no changes were detected in either TGF-*β* staining, AMPK or Erk1/2 phosphorylation levels, or in IL-1*β* expression levels. This is consistent with a potentially reversible early insult that could possibly progress into more permanent neuronal apoptosis and damage [63].

As such, we proceeded to assess several possible approaches to reverse CAN in our model. A cardioprotective role for antidiabetic drugs beyond glucose lowering is being addressed with potential emphasis on their anti-inflammatory effects [65]. Metformin and pioglitazone were selected based on the previous studies showing their cardioprotective outcomes. In the Diabetes Prevention Program, metformin significantly reduced the myocardial infarction events in prediabetic patients and this effect persisted for a 10-year follow-up [19, 66]. Furthermore, the IRIS trial found that patients with insulin resistance receiving pioglitazone had a reduced risk of stroke or myocardial infarction [67]. Recent studies suggested that metformin has a direct anti-inflammatory action independent of improvement of metabolic parameters (hyperglycemia and insulin resistance) [23]. Through activating AMPK-dependent and AMPK-independent pathways, metformin inhibits multiple inflammatory cytokines that are believed to be responsible for the potential diabetes mellitus-independent cardioprotective [68] and neuroprotective anti-inflammatory activity [69]. Similarly, pioglitazone exerts an anti-inflammatory effect through binding to PPAR receptors leading to a reduced inflammatory cytokine production accounting for its cardioprotective and neuroprotective effects [70]. The presence of saturated fat in HC diet allows for the potential association of adipose inflammatory pathways with the observed deleterious effects. Studies showed inflammatory changes in adipose tissue in response to high-fat feeding [45, 47, 71]. Indeed, in the present study, an increased expression of IL1-*β* and TNF-*α* was observed in adipose tissue of HC-fed rats consistent with prior research [43–45]. Significantly, pioglitazone [72] and metformin [73] were reported to reduce adipose inflammation. To this end, the effect of metformin and pioglitazone treatment in HC-fed rats was examined in this study. Both drugs were used in doses equivalent to the low end of those used in humans with the intent of having a minimal impact on blood glucose levels [24, 74]. The lack of an effect on blood glucose level was confirmed by regular random blood glucose measurement and OGT at the end of the treatment period.

As expected, no significance differences in noninvasive BP parameters and echocardiography were noted in metformin- or pioglitazone-treated rats. However, a trend towards a reduction of the increased vasopressor response to PE was evident for rats treated with metformin and pioglitazone. This is in agreement with findings from ex vivo experiments on isolated vessel preparations [20]. Both agents allowed recovery of BRS to values similar to those seen in control rats. In addition, time domain and frequency domain parameters recovered in rats treated by metformin or pioglitazone. Thus, the observed CAN recovery occurred independent of changes in blood glucose levels. Of note, the corrective effects observed in this study are mirrored by similar effects in diabetic patients. Studies investigating the effect of metformin on CAN in type 2 diabetic patients showed improved sympathovagal balance and HRV parameters [26, 75]. Similar results were also reported for pioglitazone [26, 76]. However, the recovery observed in the present study extends to the myocardial molecular derangement. Treatment with metformin or pioglitazone reversed the alterations in AMPK and Erk1/2 phosphorylation, together with TGF-*β* expression. As well, the observed increase in IL-1*β* and TNF-*α* adipose expression was attenuated by either treatment implying a positive impact on adipose inflammation. The putative anti-inflammatory effect extended to the reduction of the elevated cardiac IL-1*β* expression level observed in HC-fed rats.

Further evidence to support the potential association between adipose inflammation and CAN and to differentially assess the effect of high-calorie intake versus the nature of calorie source was obtained from the examination of two additional treatment groups. In the first group, the introduction of NC reduced the vasopressor response to PE and restored BRS. Whereas lowering the calorie intake while maintaining a high-saturated fat content of the chow elicited the same increased vasopressor response to PE seen in HC-fed rats and did not restore BRS. Time domain and frequency domain parameters were only restored by switching to normal chow (NC) and not lowering the calorie intake (HC-L). Consistent with these results, a previous study found that excess dietary fat, but not excess calories, increased inflammatory signals in hypothalamus which were attenuated by low-fat diet [46]. A potential link with CAN could be established considering the effect of adipose hormones on central neuronal activity. Among the adipokines, adiponectin and nesfatin have been found to exert metabolic and cardiovascular regulatory functions within the hypothalamic paraventricular nucleus [77]. Hence, it would be reasonable to assume that an alteration in the adipokine profile caused by adipose inflammation would have an impact on central neuronal function. In this regard, a neuroprotective mechanism for metformin or pioglitazone through normalizing the adipokine profile becomes plausible. While metformin is reported to penetrate blood-brain barrier [78], this ability remains highly questionable for pioglitazone [79]. Thus, in light of the absence of molecular changes consistent with signaling derangement and/or inflammation (apart from increased oxidative stress) in the brainstem compared to peripheral tissue, adipokine normalization becomes the more likely mechanism. Yet, this remains to be confirmed in future studies.

A potential limitation of the present study is the reduction of dietary protein intake due to displacement with the increased fat content. Gram for gram, the HC diet used in the present study offers 35% less protein than the normal chow. While major electrolytes were replaced, the displacement of dietary protein by refined constituents poses the added challenge of decreased intake of micronutrients including phosphorous [80, 81]. Dietary phosphorous is of particular relevance to the results of the present study given recent reports identifying its roles in regulating body weight [82], energy metabolism [83], postprandial lipemia [84], and glucose tolerance [85], implicating a potential effect on adipose tissue. Whether the results observed in this study are a direct consequence of increased dietary fat or an indirect response to micronutrient deficiency triggered by the refined diet remains to be established in future research.

In conclusion, the present study implicates the development of CAN and detrimental signaling changes in the myocardium as early consequences of mild metabolic challenge despite the normal gross cardiac structure/function and absence of signs of diabetes or impaired glucose tolerance. These early changes are likely the result of adipose inflammation and respond to interventions targeting this process. Our results highlight the importance of lifestyle changes involving reduction of the dietary content of saturated fat and provide novel insight regarding the implications of the anti-inflammatory action of metformin and pioglitazone. Future research would be required to elucidate a number of lingering questions. The potential effect of HC feeding and/or micronutrient deficiency on the adipokine profile needs to be systematically addressed. Whether an alteration in the relative abundance of different adipokines is related to the discrepant neuronal function and whether that ties into the early development of CAN remains to be determined. The question remains whether drugs like metformin and pioglitazone, and potentially statins, would exert a normalizing effect on the disrupted adipokine profile. On the other hand, without direct testing of blood-brain barrier penetration of metformin or pioglitazone, a direct neuronal effect cannot be ruled out. And finally, gender differences in the cardiac autonomic response to HC feeding requires examination together with the potential detrimental effect on the offspring.
